# Not Just a Cold Sore: Postpartum Herpes Simplex Virus Endometritis

**DOI:** 10.1155/crog/7459192

**Published:** 2026-06-11

**Authors:** Olivia Moore, Jesse Cottrell

**Affiliations:** ^1^ Department of Obstetrics and Gynecology, Marshall University Joan C. Edwards School of Medicine Medical Center Drive, Huntington, West Virginia, USA

## Abstract

Endometritis is a well‐described cause of postdelivery morbidity for both mother and neonate. Although the usual causative organisms are bacteria, in very rare cases it can be due to herpes simplex virus (HSV). In postpartum endometritis unresponsive to conventional treatment, prompt diagnosis and early antiviral therapy are required for clinical improvement in mothers and their neonates.

## 1. Introduction

Postpartum endometritis is the leading cause of febrile complication following delivery, with its incidence increasing after cesarean sections [1]. In addition to maternal fever, postpartum endometritis presents with uterine tenderness, purulent or foul‐smelling lochia, maternal tachycardia, and leukocytosis; early onset disease occurs within 48 h of delivery, and late onset disease can present up to 6 weeks postpartum [2]. Postpartum endometritis is often attributed to a polymicrobial bacterial infection, commonly due to group B streptococci, anaerobic gram‐positive cocci, and anaerobic gram‐negative Bacilli [3]. Risk factors for postpartum endometritis include cesarean section after prolonged rupture of membranes, chorioamnionitis, bacterial vaginosis, multiple vaginal examinations, the use of internal fetal monitoring, and the lack of prenatal care [4]. Treatment of uncomplicated postpartum endometritis is intravenous clindamycin plus gentamicin [2]. Rarely, however, persistent fever following antibiotics may be due to herpes simplex virus endometritis, which requires prompt evaluation and treatment [5].

## 2. Case Presentation

A 23‐year‐old female gravida 1 para 1 presented to the intensive care unit (ICU) from an outside hospital after developing postpartum sepsis 3 days after the birth of her baby. The pregnancy was complicated by prolonged rupture of membranes (21 h), maternal THC (delta‐9‐tetrahydrocannabinol) use, and polyhydramnios (self‐resolved). Prior to transfer, the patient had a primary cesarean section on hospital Day 3 due to cephalopelvic disproportion at 39 weeks and 5 days, and was diagnosed with a urinary tract infection after surgery. She received nitrofurantoin but subsequently developed an intractable fever of 103.1 F, rigors, and fundal tenderness. She was diagnosed with endometritis and given ampicillin, clindamycin, and gentamicin but continued to be febrile. The patient′s computed tomography (CT) scan of her abdomen and pelvis at the outside facility was unremarkable. Her CT pulmonary embolism study was also negative, as well as blood and urine cultures. White blood cell count was 8.7 k/cmm. Her antibiotic regimen was then switched to ceftriaxone, clindamycin, and gentamicin but fevers persisted.

The patient and her neonate were then transferred to our facility on hospital Day 6. Upon presentation to our hospital, her primary complaint was worsening tenderness of her cesarean section scar. She was febrile to 103.0 F, and the patient was transitioned to vancomycin and piperacillin/tazobactam. The patient was evaluated at bedside by the obstetrics team and was found to be awake and oriented, vitally stable, with severe abdominal and fundal tenderness. Her WBC was 5.46 k/cmm, procalcitonin was 6.3 ng/mL, lactic acid was 1.1 mmol/L, hemoglobin was 9.7 gm/dL, and hematocrit was 29.3%. A CT scan of the abdomen and pelvis was repeated which was negative for acute changes. Repeat CT pulmonary embolism study showed bilateral pleural effusions and bilateral dependent atelectasis with no significant infiltrates. On hospital Day 7, the patient′s hemoglobin was 7.1 gm/dL, and two units of packed RBCs were transfused. Her repeat blood and urine cultures had no growth. On hospital Day 8, the infectious disease team was consulted, and initial recommendations included amoxicillin‐clavulanate 875 mg BID and doxycycline 100 mg BID for 5 days.

The neonate was failing to thrive and was found to have HSV‐1 on cerebrospinal fluid analysis in the neonatal ICU, in addition to herpetic lesions on the head. This prompted virologic testing of our patient (on hospital Day 8), who notably denied a history of HSV, cold sores, or genital lesions. The patient′s serology for HSV was IgG‐negative; IgM antibodies were not obtained. The patient was discharged on hospital Day 9. Her DNA PCR for HSV‐1 from a vaginal swab was positive the next day, leading us to believe this was a primary infection that led to postpartum endometritis. Valacyclovir 1 g twice per day for 10 days was added once PCR positivity resulted for HSV‐1. The patient followed up in our outpatient clinic 2 weeks postpartum and was doing well on valacyclovir. The neonate was ultimately discharged on acyclovir after an extensive hospital stay.

## 3. Discussion

There are sparse reports of postpartum HSV endometritis cases documented in the literature. However, it remains an important cause to consider in the differential diagnosis of those with persistent postpartum fevers as diagnosis and proper management can prevent maternal and neonatal morbidity and mortality. Of the documented cases, the mainstay of treatment was with acyclovir. In 1997, Hollier et al. [6] was the first to report the death of two infants from disseminated HSV infection after being born to mothers with refractory postpartum fevers, whose endometrial viral cultures were both positive for HSV. Anyebuno et al. [7] reported two cases of prolonged maternal postpartum fever, whose infants were also failing to thrive. Ultimately, both infants′ cerebrospinal fluid HSV PCR results were positive, and one neonate died from overwhelming infection, despite initiation of acyclovir therapy. These cases highlight the importance of rapid recognition and appropriate intervention. McGill et al. [8] described a case of postpartum HSV endometritis and disseminated infection in both the mother and neonate, whose symptoms responded promptly to antiviral therapy. Remadi et al. [9] described a case of herpetic endometritis clinically manifested by delayed maternal postpartum bleeding requiring endometrial curettage and a full‐term baby that was normal. Hence, the presentation of the mother and neonate is variable, which can delay the diagnosis. Of note, most patients have no history of HSV, supporting primary infection as the culprit of postpartum endometritis in many cases. Neonatal infection is often suggestive of primary disease, with the risk of vertical transmission being 33%, compared to 3% for mothers who have reactivation of the virus [8]. HSV infection should be suspected in neonates with clinical signs of disease and whose mother has postpartum fever, unresponsive to antibiotics [7].

Experts in reproductive infectious disease are beginning to advocate for routine cervical and endometrial cultures for HSV in patients with postpartum fever that fail to respond to conventional treatment [3]. Prompt recognition and early antiviral therapy is required for clinical improvement in mothers and their neonates.

## Funding

No funding was received for this manuscript.

## Ethics Statement

No IRB was needed for this case report. Patient consent was obtained.

## Conflicts of Interest

The authors declare no conflicts of interest.

## Data Availability

Data sharing not applicable to this article as no datasets were generated or analyzed during the current study.
